# T = 4 Icosahedral HIV-1 Capsid As an Immunogenic Vector for HIV-1 V3 Loop Epitope Display

**DOI:** 10.3390/v10120667

**Published:** 2018-11-26

**Authors:** Zhiqing Zhang, Maozhou He, Shimeng Bai, Feng Zhang, Jie Jiang, Qingbing Zheng, Shuangquan Gao, Xiaodong Yan, Shaowei Li, Ying Gu, Ningshao Xia

**Affiliations:** 1 State Key Laboratory of Molecular Vaccinology and Molecular Diagnostics, School of Public Health, Xiamen University, Xiamen 361102, China; 32620150150145@stu.xmu.edu.cn (Z.Z.); maozhouhe@163.com (M.H.); 32620141150560@stu.xmu.edu.cn (F.Z.); abing0811@xmu.edu.cn (Q.Z.); gaoshuangquan@xmu.edu.cn (S.G.); xiaodongyan2008@gmail.com (X.Y.); nsxia@xmu.edu.cn (N.X.); 2 National Institute of Diagnostics and Vaccine Development in Infectious Disease, School of Life Sciences, Xiamen University, Xiamen 361102, China; 21620151153242@stu.xmu.edu.cn (S.B.); jiangj1202@163.com (J.J.); 3 Department of Chemistry and Biochemistry and Division of Biological Sciences, University of California-San Diego, San Diego, CA 92093-0378, USA

**Keywords:** HIV-1 capsid, T = 4 icosahedral, assembly, cryo electron microscopy, epitope display

## Abstract

The HIV-1 mature capsid (CA) assumes an amorphous, fullerene conical configuration due to its high flexibility. How native CA self-assembles is still unclear despite having well-defined structures of its pentamer and hexamer building blocks. Here we explored the self-assembly of an engineered capsid protein built through artificial disulfide bonding (CA N21C/A22C) and determined the structure of one fraction of the globular particles. CA N21C/A22C was found to self-assemble into particles in relatively high ionic solutions. These particles contained disulfide-bonding hexamers as determined via non-reducing SDS-PAGE, and exhibited two major components of 57.3 S and 80.5 S in the sedimentation velocity assay. Particles had a globular morphology, approximately 40 nm in diameter, in negative-staining TEM. Through cryo-EM 3-D reconstruction, we determined a novel T = 4 icosahedral structure of CA, comprising 12 pentamers and 30 hexamers at 25 Å resolution. We engineered the HIV-1 V3 loop to the CA particles, and found the resultant particles resembled the morphology of their parental particles in TEM, had a positive reaction with V3-specific neutralizing antibodies, and conferred neutralization immunogenicity in mice. Our results shed light on HIV CA assembly and provide a particulate CA for epitope display.

## 1. Introduction

The human immunodeficiency virus type 1 (HIV-1) Gag precursor protein is the major structural protein coded by the *gag* gene. During maturation, Gag is cleaved into three major structural proteins—matrix (MA), capsid (CA) and nucleocapsid (NC)—and undergoes a dramatic morphological rearrangement [1,2]. The CA protein contains two independent and highly helical domains, the N-terminal domain (NTD) and C-terminal domain (CTD), which are connected by a short flexible linker [3]. The structures of CA and its isolated domains have been solved by X-ray crystallography and nuclear magnetic resonance (NMR) spectroscopy [3,4,5,6,7]. The HIV-1 capsid has an inherent structural variability that facilitates its spontaneous assembly into different conformations in vitro [8]. However, due to the weak interactions between monomers in the pentamers and hexamers, it is difficult to obtain metastable complexes for examination. In 2010, Pornillos et al. [9,10,11] adopted a disulfide crosslinking strategy to stabilize and crystallize the soluble HIV-1 CA pentamers and hexamers, which enabled the construction of an atomic model for the complete capsid. This was accomplished via two steps: First, the pentamer and hexamer were stabilized by engineering disulfide cross-links (N21C/A22C and A14C/E45C) between the NTDs; and second, mutations (W184A/M185A) were introduced to disrupt the CTD-CTD dimeric interface that prevented the polymerization of particles [9].

The HIV-1 mature capsid resembles a fullerene cone with the hexameric lattice capped by seven pentamers at its wide end and five at its narrow end [12]. In the mature capsid, there are three different interfaces for CA–CA interactions: (i) the NTD–NTD interface between NTD domains in the hexamers; (ii) the NTD–CTD interface between the NTD and CTD domains belonging to neighboring subunits of the same hexamer; (iii) the CTD–CTD interface between CTD domains belonging to neighboring hexamers [13]. Advancements in cryo-electron microscopy (Cryo-EM) accelerated the structural determination of the HIV capsid, and a recent study reported the subnanometer structural resolution of hexameric and pentameric CA within intact HIV-1 particles by cryo-electron tomography (Cryo-ET) [14]. The hexamer structure is compatible with previous crystallography studies but the pentamer forms by means of different interfaces [14]. Given the important function of the HIV-1 capsid in the virus life cycle, CA has become a promising target for the development of anti-HIV-1 inhibitors [15,16,17,18]. Recently, Dick et al. [19] reported that inositol phosphates are assembly co-factors for HIV-1 that bind to highly conserved sites in CA. An investigation of CA structure will help to reveal the virion assembly mechanism and accelerate the development of novel anti-HIV-1 drugs targeting virion assembly.

A HIV-1 vaccine is thought to be an ideal way to prevent HIV-1 infection, but such a vaccine is still “on the way”. In recent years, dozens of HIV-1 broadly neutralizing antibodies (bNAbs) have been isolated from the HIV-1 infected individuals, which mainly target the V1V2 loop, V3 loop, CD4 binding site, fusion peptide (FP), gp120–gp41 interface, and membrane proximal external region (MPER) of HIV-1 Envelop (Env) [20,21]. Some epitope structures of these bNAbs have been determined to guide the design of better immunogens [22], and some of the bNAbs have been subjected to clinical trial to test their potential for prevention and therapy of HIV-1 [23,24,25]. Numerous strategies were developed for bNAbs elicitation, of which the use of heterologous scaffolds to present specific broad-neutralization epitopes has garnered much interest. Recently, Xu et al. designed the FP-bearing immunogens and conducted the iterative structure-based optimization to elicit the FP specific antibodies, which provides proof of principle to generate the bANbs by epitope-based approach [26]. The encouraging progress of bANbs will accelerate the development of an efficacious HIV-1 vaccine. Furthermore, it is widely accepted that both bNAb elicitation and cell-mediated immune responses are crucial for a successful HIV-1 vaccine [27,28]. In clinical studies of vaccine efficacy, studies have noted that Gag induces potent T-cell responses [29,30,31]. Given that CA is a proteolytic product of Gag, CA may offer a useful scaffold to fuse Env antigens in immunogen design. 

In this study, we analyzed a previously engineered a disulfide, cross-linking capsid protein (named CA N21C/A22C) that assembled in vitro, and solved its structure as a novel T = 4 icosahedral particle. CA N21C/A22C was then used to display the HIV-1 V3 loop, and tested for its immunization potential in BALB/c mice. These results will provide significant information for the further screening of antivirals associated with capsid assembly and in the development of epitope vaccines based on a capsid scaffold.

## 2. Materials and Methods

### 2.1. Plasmid Construction and Protein Purification

The HIV-1 wild type (WT) capsid CA gene was cloned from coding regions of HIV-1 NL4-3. The CA sequence was amplified by PCR and cloned into the pMD 18-T vector using a TA cloning kit (Takara, Dalian, China). CA was then cloned into the pTO-T7 expression vector, and expression confirmed by DNA sequencing. The CA N21C/A22C mutant was generated with the QuickChange Lightning Site-Directed Mutagenesis Kit (Agilent, Santa Clara, CA, USA) and verified by DNA sequencing. The CA N21C/A22C mutant was transformed into *Escherichia coli* BL21 (DE3) for protein expression. The mutant protein was expressed by IPTG for 12 h at 25 °C. Cell pellets were re-suspended in 50 mM Tris-HCl buffer, pH 8.0, 50 mM NaCl, 200 mM βME and sonicated. The supernatant was precipitated with 25% ammonium sulfate and further purified using an SP-Sepharose column (GE Healthcare, New York, NY, USA).

The chimeric CA N21C/A22C-V3 protein was constructed and purified as described above. Residues A88 and R97 of the cyclophillin binding loop were replaced with the NL4-3 V3 loop sequence (TRPNNNTRKSIRIQRGPGRAF VTIGKIGNMRQAH, without terminal cysteines C294 and C329).

### 2.2. Protein Assembly In Vitro, SDS–PAGE and Western Blotting (WB)

Purified protein (1 mg/mL) was dialyzed into assembly buffer (50 mM Tris-HCl, pH 8.0, 1 M NaCl) overnight at 4 °C. Proteins were separated on 12% polyacrylamide gels (with 5% acrylamide in the stacking gel). For reducing conditions, proteins were resuspended with 2 × loading buffer (100 mM Tris-HCl, pH 6.8, 0.1% SDS, 0.2% bromophenol blue, 200 mM βME and 20% glycerol). For non-reducing conditions, the same buffer was used without βME. For WB, separated proteins were transferred to nitrocellulose membranes, blocked with 5% fat-free milk in Tris-buffered saline, and incubated for 1 h with 1 µg/mL anti-CA mAb A10F9 [32] at room temperature. Subsequently, the membranes were washed five times with PBS (pH 7.4) containing 0.2% Tween-20 (PBST) and incubated with HRP-conjugated goat anti-mouse secondary antibody (1:5000 dilution). After washing in PBST, color development was performed with 3, 3′, 5, 5′-tetramethylbenzidine (TMB) substrate.

### 2.3. Size-exclusion Chromatography, Analytical Ultracentrifugation (AUC), and Transmission Electron Microscopy (TEM)

Size-exclusion chromatography was performed on an Agilent 1200 HPLC (Agilent Technologies, Inc., CA, USA). Assembled particles were analyzed on a G5000PWxl GC column (TOSOH, Tokyo, Japan) in assembly buffer at a 0.5 mL/min flow rate. The 280 nm wavelength UV absorbance was recorded.

Sedimentation velocity (SV) was used to evaluate the sedimentation coefficient and molecular size of CA and CA N1C/A22C before and after assembly. AUC experiments were performed at 20 °C on a Beckman XL-A analytical ultracentrifuge (Beckman Coulter, Fullerton, CA, USA) equipped with an absorbance optics and an An60-Ti rotor. Test samples were diluted to a final concentration of 1 mg/mL. Data were processed with Sedfit software (Center for Information Technology, NIH, Maryland, USA) to obtain the sedimentation coefficient and molecular size.

TEM was performed on an FEI Tecnai G2 Sprit electron microscope operating at 120 keV. For sample preparation, an 8-μL aliquot of assembled protein (0.1 mg/mL) was adsorbed to a carbon-coated grid for 5 min. Filter paper was used to remove excess liquid, and the grid was stained with 5% phosphotungstic acid. Particles were selected, and their diameters measured using the IPWIN Application 6.0 software (Media Cybernetics Inc., Sarasota, FL). The distribution of diameters was calculated using the GraphPad Prism software (GraphPad Prism, San Diego, CA, USA). 

### 2.4. CryoEM Data Collection and Structure Determination

Aliquots (3 µL) of the purified sample were deposited onto a glow discharged holey carbon Cu grid (R2/2, 200 mesh, Quantifoil Micro Tools). Excess liquid was removed by blotting for 6 s before plunge-freezing in liquid ethane cooled by liquid nitrogen inside an FEI Mark IV Vitrobot (Thermo Fisher Scientific, Carlsbad, CA, USA). The cryoEM grids were viewed in an FEI Tecnai F30 TEM (Thermo Fisher Scientific, Carlsbad, CA, USA) at 300 kV, equipped with an FEI Falcon II direct detector camera. Data were collected at 93,000 nominal magnification (pixel size of 1.128 Å) with the defocus ranging from 1.0 to 3.0 µm underfocus using EPU automatic data collection software (FEI). The total electron dose was 25 e^-^/Å^2^, with an exposure time of 1 s. A total of 958 micrographs were obtained for further processing. Drift correction and CTF estimation were processed with Motioncorr2 [33] and Gctf [34]. Particles (9766) were manually selected using the e2boxer.py program in the EMAN 2.1 package [35]. An initial 3D model was generated with the random model method by AUTO3DEM [36]. Several rounds of reference-free 2D classification and 3D reconstruction were implemented. Then, favorable particles were selected for further 3D refinement using 3D auto-refine in RELION 1.4 [37], where two models are refined independently. As no symmetric information could be acquired from the sample, we tried different symmetric manners for reconstruction, including I2, I3, and I4, to refine the density map. The final resolution estimation was based on the gold standard Fourier shell correlation with a threshold criterion of 0.143. Visualization, map generation, and dihedral angle analysis were conducted with Chimera [38] and PyMol [39].

### 2.5. Ethics Statement and Mice Immunization

Animal experiments were approved by the Xiamen University Laboratory Animal Center (Approval number: XMULAC20160051; approval date: 07/03/2016). All procedures were conducted in accordance with animal ethics guidelines and approved protocols. Five female BALB/c mice (6 weeks old) were immunized using an intraperitoneal injection of 50 µg CA N21C/A22C-V3 protein, formulated with aluminum adjuvant. The immunization scheme had six dosages, given at 2-week intervals. Pre-immune and post-immune sera (week 8, 10 and 12) were collected, heat-inactivated at 56 °C for 30 min, and stored at −20°C for further analysis. Three female BALB/c mice were injected with CA N21C/A22C protein as a negative control following the same immunization schedule.

### 2.6. Enzyme-Linked Immunosorbent Assay (ELISA)

Proteins (100 ng/well) or peptides (200 ng/well) were coated into the wells of 96-well microplates at 37 °C for 2 h. The plates were then blocked with blocking buffer at 37 °C for 2 h. After five washes, 100 μL of serially diluted monoclonal antibodies or antiserum were added into the wells and incubated at 37 °C for 1 h. The plates were then washed and incubated with 100 μL horseradish peroxidase (HRP)-conjugated goat anti-mouse secondary antibody (1:5000 dilution) at 37 °C for 30 min. Color was developed with 100 μL 3,3′,5,5′-tetramethylbenzidine was added to develop the color. The reaction was terminated with 2 M H_2_SO_4_, and the OD value was measured at 450 nm and 630 nm using a microplate reader.

### 2.7. HIV-1 Neutralization Assay

TZM-b1 cells (1 × 10^4^ cells/well) were grown in 96-well plates in 100 μL of Dulbecco’s Modified Eagle’s Medium supplemented with 10% fetal bovine serum and cultured overnight. The next day, the 50% tissue culture-infective dose (TCID_50_) of HIV-1 containing 15 μg/mL of DEAE-dextran (50 μL/well) was mixed in a tube with 20-fold diluted antiserum (100 μL/well), and incubated at 37 °C for 1 h. The mixtures were then added to the cells and incubated at 37 °C for 48 h. Cells were then fixed with 0.2% glutaraldehyde and stained with X-gal substrate. HIV-1-infected cell spots were counted using an Immunospot Series Analyzer (Cellular Technology, Cleveland, OH, USA). The percent neutralization (%) was calculated as: [1−(spots in immunization antiserum-treat wells / spots in pre-immunization antiserum-treat wells)] × 100.

## 3. Results

### 3.1. Purification and Characterization of HIV-1 CA N21C/A22C

**Figure 1:**
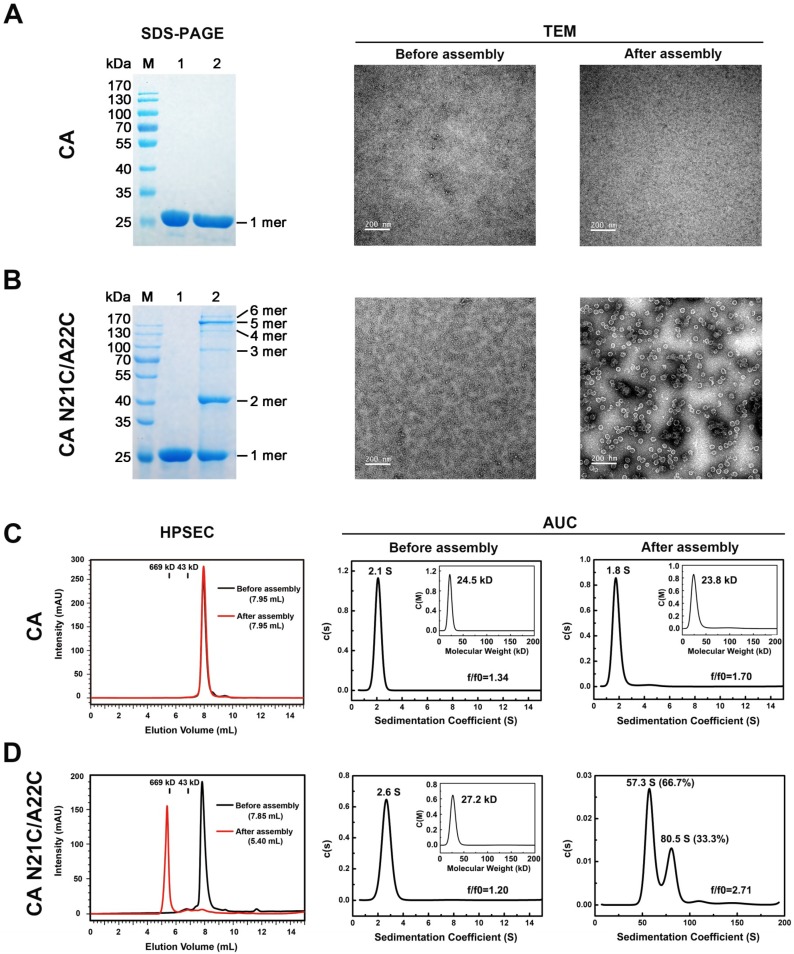
Characterization of CA N21C/A22C assembly in vitro. (**A**) CA N21C/A22C self-assembly was analyzed with non-reducing SDS-PAGE and negative staining transmission electron micrography (TEM). Lane M, molecular weight marker; lane 1, the protein before assembly; lane 2, the protein after assembly in the buffer (50 mM Tris-HCl, pH 8.0, 1 M NaCl). The positions of cross-linked n-mers are indicated on the right. The EM image scalebar is 200 nm. (**B**) Same analysis as in (**A**) for the wild-type CA as a control. There is no observable assembly for WT CA under the same conditions. (**C**) Self-assembly of CA N21C/A22C, analyzed by size-exclusion chromatography (HPSEC) and analytical ultracentrifugation (AUC). The retention times of the protein standards are indicated as black lines (numbers indicate molecular mass in kilodaltons). The sedimentation coefficient and apparent molecular size were measured by c(s) and converted c(M) method in the sedimentation velocity experiment. The f/f0 indicates the hydrated friction ratio. (**D**) Same analysis as in (**C**) for wild-type CA as a control. There is no observable assembly for WT CA under the same conditions.

The pure CA N21C/A22C protein was dialyzed into assembly buffer (50 mM Tris-HCl, pH 8.0, 1 M NaCl) overnight at 4 °C. In the subsequent experiments, we characterized CA N21C/A22C before and after assembly and compared with WT CA. WT CA presented as a monomer in SDS-PAGE both before and after dialysis, with no particles in negative-staining EM images (Figure 1A). As expected, the CA N21C/A22C cross-linked into polymers after assembly compared to before assembly, as shown on SDS-PAGE (Figure 1B). It is important to note, however, that we observed a 6-mer band in the non-reduced SDS-PAGE, which is inconsistent with the results reported by Pornillos et al. [9] where CA N21C/A22C assembled at 10 mg/mL in assembly buffer (50 mM Tris-HCl, pH 8.0, 1 M NaCl and 20 mM β-ME) and resolved only with 5-mer bands and no 6-mer bands in gel. To confirm our results, we used a higher starting concentration of CA (approximately 4 mg/mL and 10 mg/mL) in the self-assembly and found that the presence of hexamers in non-reduced SDS-PAGE was independent of the starting protein concentrations (Figure S1A). The negative staining result of the assembled protein suggested that CA N21C/A22C assembled into heterogeneous, spherical particles (Figure 1B), most with a diameter of ~40 nm (Figure S1B).

Next, we analyzed the assembly of CA N21C/A22C by HPSEC and AUC. In the HPSEC analysis, we found that the elution volume was approximately 7.85 mL and 5.40 mL for CA N21C/A22C before and after assembly, respectively (Figure 1D). However, an elution volume of 7.95 mL was measured for WT CA both before and after assembly (Figure 1C). Meanwhile, through AUC, we found that the assembled CA N21C/A22C exhibited two major peak values, with sedimentation coefficients of 57.3 S (66.7%) and 80.5 S (33.3%), respectively, as compared to 2.6 S (monomer in solution according to molecular weight) before assembly. WT CA, on the other hand, had similar sedimentation coefficients (~2.1 S) before and after assembly, corresponding to its monomeric form under both conditions (Figure 1A,C). Taken together, these results show that CA N21C/A22C self-assembles into particles in relatively high ionic solutions.

### 3.2. Three-Dimensional Reconstruction of HIV-1 CA N21C/A22C Particles

**Figure 2:**
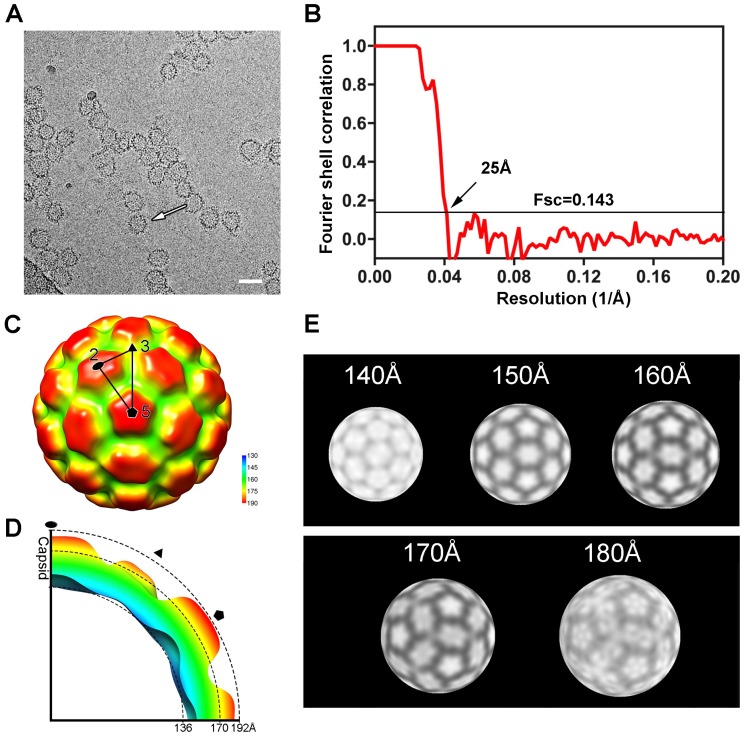
Cryo-electron micrography (Cryo-EM) and three-dimensional reconstruction of the CA N21C/A22C particle. (**A**) A representative cryo-electron micrograph of the CA N21C/A22C particle (white arrow). Scalebar is 50 nm. (**B**) Gold-standard Fourier Shell Correlation (FSC) curve for reconstruction of the N21C/A22C particle. (**C**) Surface rendering colored radially (blue to red) of the CA N21C/A22C cryo-EM density map viewed along a five-fold axis. The two-, three- and five-fold symmetry axes are indicated by the black ellipse, triangle and pentagon, respectively. An asymmetric unit (indicated by the big triangle) contains four CA subunits: One from a pentamer and three from a hexamer. (**D**) Density section of the N21C/A22C indicated in black dash lines with symmetry axes labeled. The inner and outer radii of capsid are 136 Å and 192 Å respectively, along the 2-fold axis. (**E**) Different radial projections of the map at the different radii (140 Å to 180 Å). The three hexamers show interactions in the range of 160Å to 180Å.

Three-dimensional reconstruction of the cryo-EM structure of the CA N21C/A22 particle was performed. Protrusions of the assembly capsids were observed in the cryo-EM micrographs, but the particles exhibited high heterogeneity (Figure 2A). After 13 rounds of 2D classifications using RELION software (https://www2.mrc-lmb.cam.ac.uk/relion/index.php) (Figure S2), we reconstructed a representative structure of the CA N21C/A22 particle using the most-homogeneous particle in the major class. As for 3D classification and reconstruction, an initial model was generated by the random model method using AUTO3DEM software [36], and subjected to final reconstruction in RELION [37]. Of note, it is not adequate for this dataset, which contains so few particles, to be used to implement a structure reconstruction while imposing C1 symmetry to the calculation. Thus, we applied every available icosahedral (I) symmetry including I2, I3 and I4, for 3D reconstruction to ascertain the most reasonable I symmetry. We obtained an interpretable map with I2 symmetry that illustrated well-defined morphology for the pentamers and hexamers in the icosahedron (Figure S2). The structure was determined at a resolution of 25 Å by gold-standard calculations with a Fourier shell correlation coefficient (FSC) cutoff criterion of 0.143 (Figure 2B). The density map had a T = 4 icosahedral capsid structure, which consists of 12 pentamers and 30 hexamers (Figure 2C). Each asymmetric unit contained four CA subunits: one subunit from a pentamer and three from the adjacent hexamer (Figure 2C). Obvious concaves were observed around the pentamer and hexamer in the map, likely attributable to linkages among neighboring capsomers. The inner and outer radii of the capsid along the 5-fold axis were 136 Å and 192 Å, respectively (Figure 2D). Navigating along the radial central axis from inside to out, we noted disconnectivity among the electric densities attributed to the three hexamers surrounding the three-fold symmetry axes at the section spanning 140 Å to 150 Å, but visible connections among the three hexamers in the section ranging from 160 Å to 180 Å (Figure 2E); these are assumed to be key interactions for capsid assembly.

### 3.3. Inter-Subunit Interactions in Particles

**Figure 3:**
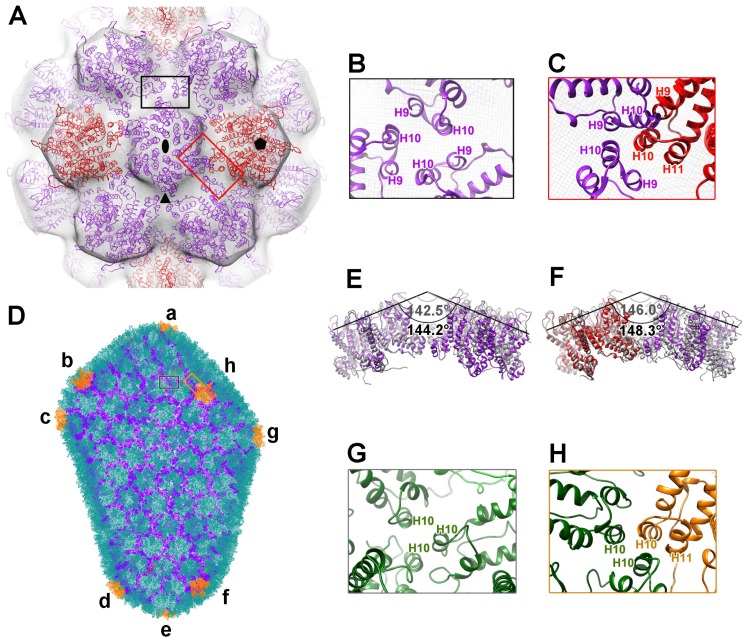
The models and major interactions of T = 4 CA N21C/A22C particle and fullerene capsid. (**A**) The atomic model of T = 4 CA N21C/A22C particle embedded in the semi-transparent cryo-EM density map, viewed along a two-fold axis (pentamer PDB no. 3P05, hexamer PDB no. 3H47). The pentamers are red, and the hexamers are purple. The two-, three- and five-fold symmetry axes are indicated by the black solid ellipse, triangle and pentagon, respectively. (**B** and **C**) Close-up views of the boxed regions in (**A**), showing the hexamer–hexamer (B, black box) and pentamer–hexamer (C, red box) interaction interfaces. (**D**) The atomic model of the fullerene capsid (PDB no. 3J3Q). Hexamers are green, pentamers are orange. Twelve pentamers were labelled a to l; pentamers i to l are hidden, as they are on the opposite side of the model. Refer to Figure S3 to observe all the pentamers. (**E** and **F**) Dihedral angles (angle between two planes defined by neighboring pentamers or hexamers) of hexamer–hexamer (**E**) and pentamer–hexamer (**F**) in the CA N21C/A22C. These dihedral angles resemble those of the pentamer labelled “h” (colored in gray). (**G** and **H**) Close-up views of the boxed region in (**D**), representing the interaction interfaces in the hexamer–hexamer (**G**) and pentamer–hexamer (H**)**.

We fitted the crystal structures of the HIV-1 CA pentamer (PDB no. 3P05) and hexamer (PDB no. 3H47) into the T = 4 cryoEM density map, and generated an atomic model for the CA-N21C/A22C particle. We identified two unique clustering interactions involving three capsomers located around a 3-fold axis and a local pseudo 3-fold axis that surrounded a 5-fold axis (Figure 3A). For the first clustering interaction among the hexamers in the atomic model, helix 9 of one hexamer makes connections with neighboring helix 10 from two adjacent hexamers (Figure 3B). This interaction, occurring at the CTD of the triple-fold clustering CAs, was previously reported to form a hydrophobic interface and play a critical role in mature capsid assembly and stability [40]. As to the second clustering interaction, helices 9 and 10 from one pentamer were assumed to interact with the same helices of two neighboring hexamers, despite some collisions in our fitted model (Figure 3C). The mismatch in the local configuration of helices 9 and 10 of the pentamer between the crystal structure and the authentic particle model could be due to the high flexibility of helices 9 and 10 of the pentamer, which may conformationally adapt during particle assembly.

We next compared the intertwined angles of the representative hexamer–hexamer and pentamer–hexamer interactions (or the dihedral angles between two planes defined by the pentamer or hexamer) for the T = 4 icosahedral association with that of the entire fullerene capsid structure generated by molecular dynamics; the structure was based on cryo-ET images of the native capsid, where 12 pentamers (labelled a–l) were introduced to the hexamer-netting conical entity [40] (Figure 3D, Figure S3A). Among the dihedral angle comparisons, the angles for the pentamer–hexamer and hexamer–hexamer with the fullerene capsid closest to that of the T = 4 particle were located nearest to pentamer h:142.5 vs. 144.2 and 146.0 vs. 148.3, respectively (Figure 3D,F, Figure S3B). Unlike the interaction elements involved in the globular particle association, only helix 10 from the hexamer and helix 10 and helix 11 from the pentamer are involved in the inter-capsomer interaction of the fullerene assembly (Figure 3G,H), which may due to the local assembling requirement for the two different capsid structures. Nevertheless, some assembled blocks of the T = 4 icosahedral lattice may represent one of the assembling intermediates of the whole fullerene capsid assembly.

### 3.4. V3 Loop Grafted to CA Particles Induces Neutralizing Antibodies

**Figure 4:**
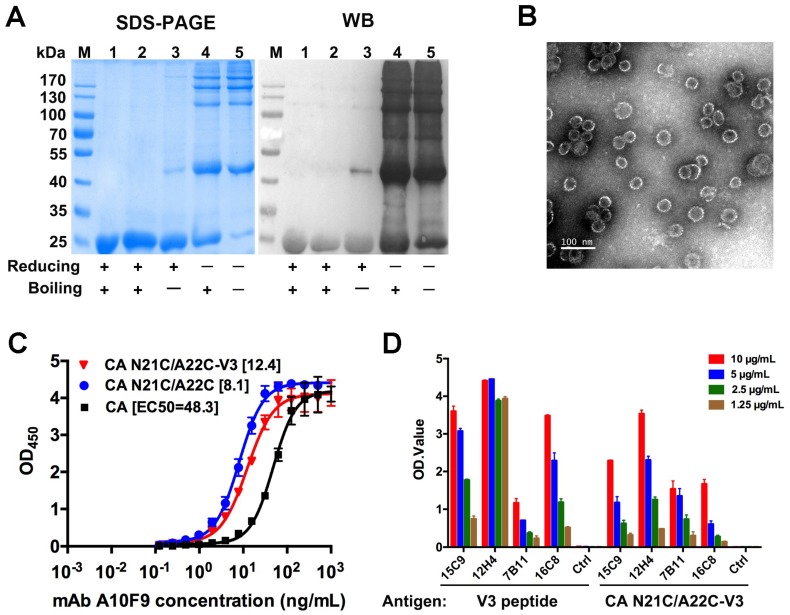
Characterization of CA N21C/A22C-V3. (**A**) SDS-PAGE and western blotting of the assembled CA N21C/A22C-V3. Lane M, molecular weight marker; lane 1, protein before assembly; lane 2-5, protein after assembly. The primary antibody is a CA specific mAb A10F9. (**B**) Negative-stained electron microscopy image of a CA N21C/A22C-V3 particle. Scalebar is 200 nm. (**C**) Antigenicity of CA N21C/A22C-V3 was determined with the A10F9 mAb using indirect ELISA. Data were analyzed by GraphPad Prism software. The 50% maximal effective concentration (EC_50_) was calculated by a four-parameter logistic fit, and is indicated in the square brackets. (**D**) Antigenicity of CA N21C/A22C-V3 particles was measured by four anti-V3 mAbs (15C9, 12H4, 7B11 and 16C8) using indirect ELISA. These mAbs were found to have specific reactivity with the NL4-3 V3 synthesized peptide. CA N21C/A22C-V3 shows good reactivities with the mAbs, indicating that the V3 loop is properly displayed on the CA N21C/A22C particle scaffold. Data are the mean and standard deviations. All experiments were repeated three times.

To investigate the potential to use CA N21C/A22C as a scaffold protein, the Env V3 loop was grafted, replacing residues A88 to R97 in the cyclophilin-binding loop of CA. The chimeric protein—CA N21C/A22C-V3—was expressed and purified as described for the parental CA N21C/A22C. After self-assembly, CA N21C/A22C-V3 presented in hexameric form in non-reducing SDS-PAGE and dissociated into monomers under reducing conditions, and showed reactivity with the CA-specific mAb (A10F9) in WB (Figure 4A). The chimeric particles bearing the V3 loop were of a similar morphology and size as CA N21C/A22C on negative staining TEM (Figure 4B and Figure 1B).

We next determined the antigenicity of CA N21C/A22C-V3 particles using an ELISA assay with the A10F9 mAb. We found that A10F9 mAb had a lower 50% maximal effective concentration (EC_50_) with the CA N21C/A22C-V3 and CA N21C/A22C than with CA (Figure 4C), which indicated that CA N21C/A22C-V3 has retained the same antigenicity as that of CA N21C/A22C. As to antigenicity of Env, CA N21C/A22C-V3 demonstrated good reactivity against a panel of HIV neutralizing mAbs including mAb 15C9, 12H4, 7B11 and 16C8, which is comparable to the reaction profiles of the corresponding V3 loop peptide (Figure 4D). Taken together, the V3 loop engrafted on the CA N21C/A22C particle scaffold did not perturb CA particle assembly and well exposed its neutralization epitope.

**Figure 5:**
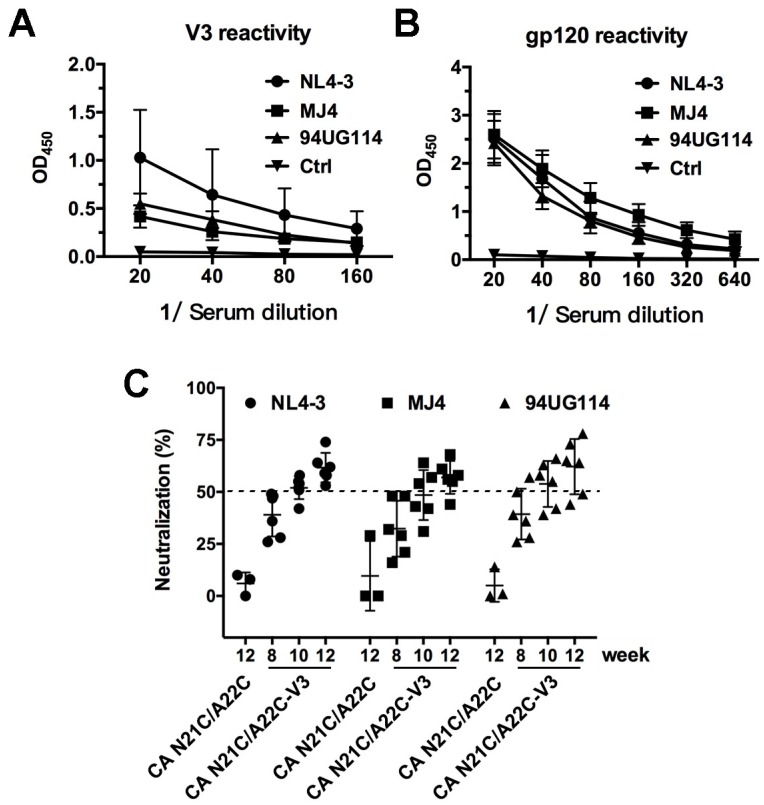
Immunogenicity of CA N21C/A22C-V3. (**A** and **B**) The reactivity of antiserum against V3 peptides (**A**) and gp120 proteins (**B**) from NL4-3 (clade B), MJ4 (clade C) and 94UG114 (clade D), as measured by ELISA. Pre-immune sera served as a control. (**C**) Neutralization assay of antiserum using HIV-1 NL4-3, MJ4 and 94UG114 viruses. Serum from the immunization of CA-N21C/A22C particle (week 12) served as a control. Data are the mean and standard deviations. All experiments were repeated three times.

Finally, we evaluated the immunogenicity of the CA N21C/A22C-V3 protein in BALB/c mice. With pre-immune serum serving as a negative control, the endpoint immunized antisera were used to evaluate reactivity to V3 synthetic peptides and gp120 proteins of three different HIV clades (clade B: NL4-3, clade C: MJ4 and clade D: 94UG114). As shown in Figure 5A,B, the antisera induced by CA N21C/A22C-V3 protein (NL4-3 strain) showed reactivity with the V3 loop and gp120 at ~160- and ~640-times dilution, respectively, and the reactivities were comparable among the three clades. A virion-based neutralization assay was used to measure the neutralizing activities of antisera against the three clades HIV-1 virus. We found that the CA N21C/A22C-V3 immunizing serum (at 20-times dilution, >50% neutralization) [41,42] neutralized all three clades with increasing neutralizing activities from week 8 to week 12. No neutralizing activity was observed in the CA N21C/A22C control group at week 12 (Figure 5C). These results suggest that CA N21C/A22C-V3 particles can efficiently elicit cross-clade neutralizing antibodies against the V3 loop.

## 4. Discussion

The HIV-1 capsid protein not only plays a crucial role in HIV-1 maturation but also in HIV-1 infection [15,43]. During virion maturation, the CA undergoes dramatic conformational rearrangements to produce the infectious virions [43]. During HIV-1 infection, CA—which houses the viral RNA genome—participates in a series of interactions with host cell factors to facilitate target cell infection [15]. Because of this important role, CA is considered an ideal target for antiviral therapy [44]. An investigation into the mechanism of CA structure and assembly could thus provide information for the development of novel antiretroviral agents. Recent reports name inositol phosphates as assembly co-factors for HIV-1, which could offer a potential avenue for the development of therapeutic strategies that target HIV-1 replication [19]. The capsid itself shows intrinsic flexibility in its assembly [8], and although the immature and mature CA structures have been resolved by Cryo-ET and Cryo-EM [14,45], an understanding of CA assembly in vitro could offer new structural insight, which would be beneficial for the understanding of CA assembly and in the development of future antiviral screening.

In this study, we analyzed a previously engineered a disulfide cross-linked capsid and revealed a novel T = 4 capsid structure that is different to that deduced by Pornillos et al. [9]. The group reported that the N21C/A22C construct showed an enriched 5-mer band in the SDS-PAGE profile, which assembled into 35-nm spherical particle. There was no band indicative of a 6-mer. These particles were speculated as T = 3 icosahedral particles, and pentamers could serve as blocks similar to hexamers in a so-called quasi-equivalent manner during particle assembly [9]. However, our results showed that the assembled N21C/A22C presented both 5-mer and 6-mer bands under non-reducing SDS-PAGE, and assumed a T = 4 icosahedral symmetry arrangement with a 40-nm diameter. We initially speculated that these discrepancies might be due to the starting protein concentration in vitro. However, our results were consistent at higher concentrations (4 mg/mL and 10 mg/mL). Thus, we proposed that the absence of reductant in assembly buffer (50 mM Tris-HCl, pH 8.0, 1 M NaCl) may be the reason for conspicuous hexameric association of CA N21C/A22C occurring in our assembly product, as compared to the assembly buffer containing 20 mM βME applied and none hexamer observed in Pornillos’ study [9]. In addition, we also observed smaller particles than T = 4 in both negative TEM (Figure S1) and cryoEM 2D classification (Figure S2), indicating other assembled form should not be completely excluded in the assembly of CA N21C/A22C in our study. Taken together, our assembly sample mostly contained a population of 40 nm particles (corresponding to T = 4 size), which showed high heterogenicity per se and consequently resist to be aligned well in molecular level for structure determination at high resolution. Nevertheless, variable morphology of heterogeneous CA N21C/A22C particles may partially reflect the inherent structural variability of the HIV-1 capsid that assembles into different conformations in vitro.

Cardone et al. [46] reported that the Rous sarcoma retrovirus (RSV) CA protein assembled into T = 1 and T = 3 icosahedrally symmetric capsids in vitro, of which T = 3 particles with 35-nm diameter showed quasi-equivalent interacting surfaces in pentamers and hexamers. The CA N21C/A22C in our study had similar interacting interfaces to the RSV CA protein. In the HIV-1 mature virions, the conical fullerene capsid of HIV-1 displays a constantly variable lattice curvature. Indeed, our CA N21C/A22C particles have similar dihedral angles as those of the pentamer–hexamer and hexamer–hexamer interactions in the fullerene model (PDB no. 3J3Q) (Figure S3). These results may indicate that CA N21C/A22C spherical particles adopt the same pentamer–hexamer and hexamer–hexamer interaction interfaces. The interaction at the three-fold and quasi three-fold interface is critical for capsid assembly regardless of the conical and icosahedral shape of the capsid. However, because of the limited resolution, we cannot interpret the precise interacting details in the CA N21C/A22C structure. In terms of the capsid structure, there are different assembly states during the maturation process. Thus, the T = 4 CA N21C/A22C particles may be similar to the potential intermediates that form during the assembly process, which could be used as part of a capsid assembly model for antiviral screening.

The HIV-1 Gag protein has been investigated in vaccine research [29], and despite the long history of HIV-1, there is still no effective vaccine to prevent HIV-1 infection. In more recent years, studies have described the potential utility of various broadly neutralizing antibodies for vaccine design, which predominantly target the V1V2 loop, the V3 loop, the CD4 binding site, and the membrane proximal external region (MPER) of HIV-1 [47]. The elicitation of these HIV-1 broadly neutralizing antibodies has been investigated through a range of different strategies, among which grafting of neutralizing epitopes onto heterologous protein scaffolds has been extensively [26,48,49,50]. HIV-1 Env V3 region plays a critical role for co-receptor binding during the infection process and is also a major target recognized by neutralizing antibodies [51]. Many attempts have been made to design V3-based immunogens [52,53,54,55,56]. Tagliamonte et al. [57] have successfully grafted HIV-1 V3 loop into CA hexamer-only tube, despite no immunization data being shown. Here, we harnessed this approach, using CA as a scaffold to fuse the Env antigen; this would not only present a neutralizing epitope for eliciting HIV-1 neutralizing antibodies but also provide a CA epitope for inducing HIV-1 non-neutralizing antibodies and stimulating antigen-specific CD8+ cytotoxic T lymphocytes (CTLs), a two-pronged strategy previously proposed in the literature as a likely requirement for the appropriate design of a HIV-1 immunogen [27,28]. We show that the NL4-3 V3 loop displayed on CA N21C/A22C retained anti-V3 mAb binding activity, suggesting that the V3 loop epitope was effectively exposed. Moreover, the chimeric CA N21C/A22C-V3 protein was able to assemble into spherical particles, suggesting that the grafted V3 loop does not interfere with particle assembly. The immunization assay showed that the chimeric CA N21C/A22C-V3 elicited a cross-clade neutralizing antibody response in BALB/c mice. Although this study has provided preliminary evidence to suggest CA N21C/A22C particle to be an immunogenic vector, future studies should explore grafting of other Env antigens onto the CA N21C/A22C scaffold to evaluate its advantages and potential applicability as a HIV-1 vaccine.

In conclusion, we obtained the T = 4 CA N21C/A22C structure at a lower resolution due to the high heterogenicity of the CA particle, and compared its dihedral angles to the conical capsid; our results suggest some similarity in the curvature between these two types of capsid assemblies. Furthermore, we found that the HIV-1 V3 loop displayed by CA N21C/A22C could induce cross-clade neutralizing antibody responses in BALB/c mice. These findings will provide important information for future antiviral designs targeting capsid assembly and for vaccine development based on the capsid scaffold.

## Supplementary Materials

The following are available online at http://www.mdpi.com/1999-4915/10/12/667/s1, Figure S1: SDS-PAGE of CA N21C/A22C assembled in three starting protein concentrations (A) and the diameter distribution of CA N21C/A22C particles (B). Figure S2: The workflow of 3D-EM reconstruction of the CA N21C/A22C particles. Figure S3. The measurement of dihedral angles of pentamer–hexamer and hexamer–hexamer in the fullerene model (PDB no. 3J3Q).
