# Supplementary material for: T = 4 Icosahedral HIV-1 Capsid As an Immunogenic Vector for HIV-1 V3 Loop Epitope Display
Source: Viruses. 2018 Nov 26;10(12):667. doi: 10.3390/v10120667 (PMC6316451; doi:10.3390/v10120667)
Supplement: Supplementary file 1 [file viruses-10-00667-s001.zip › 0-viruses-382038-supplementary/Supplemenatry files/6-viruses-382038-supplementary captions.docx]

Supplementary Captions

Figure S1: SDS-PAGE of CA N21C/A22C assembled in three starting protein concentrations (A) and the diameter distribution of CA N21C/A22C particles (B). The particles were picked and their diameters were measured using the IPWIN Application 6.0 software. The diameter distribution was calculated by GraphPad Prism software.

Figure S2: The workflow of 3D-EM reconstruction of the CA N21C/A22C particles. 958 micrographs were preprocessed, and 9766 particles were manually picked for further 2D classification. After 13 rounds of 2D classification, 722 particles were retained for 3D reconstruction, which was applied with every potential symmetry through trial and error.

Figure S3. The measurement of dihedral angles of pentamer–hexamer and hexamer–hexamer in the fullerene model (PDB no. 3J3Q). (A) The distribution of 12 pentamers in the fullerene model. (B) The dihedral angles for all inter-capsomer associations.
