# Supplementary figures and images for: T = 4 Icosahedral HIV-1 Capsid As an Immunogenic Vector for HIV-1 V3 Loop Epitope Display
Source: Viruses. 2018 Nov 26;10(12):667. doi: 10.3390/v10120667 (PMC6316451; doi:10.3390/v10120667)

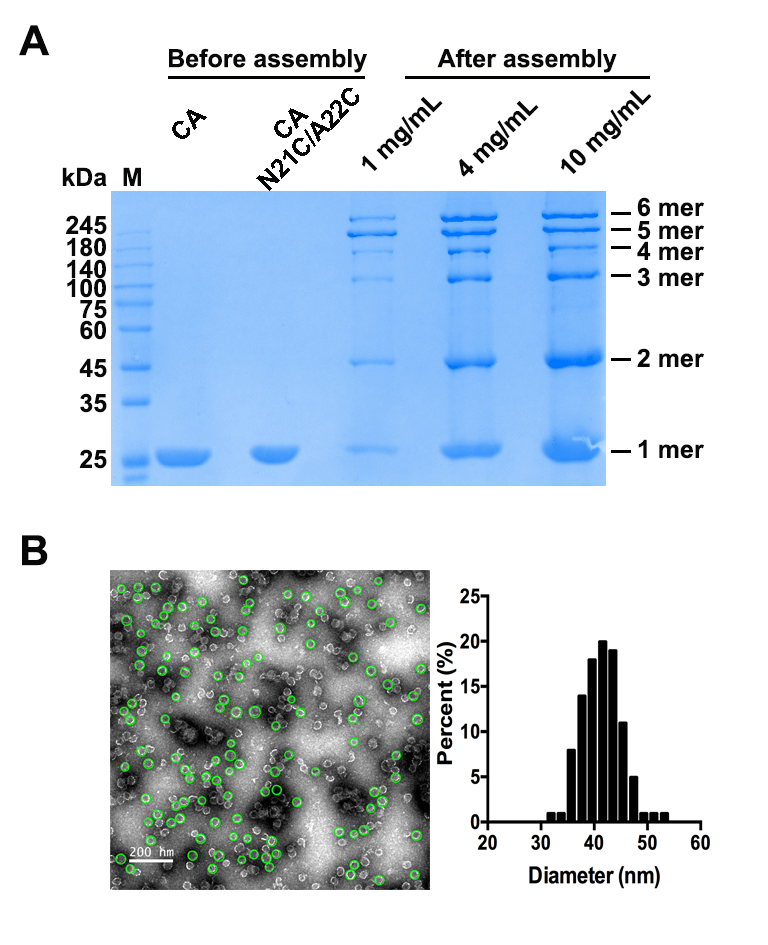

Supplement: Supplementary file 1 [file viruses-10-00667-s001.zip › 0-viruses-382038-supplementary/Supplemenatry files/Supplementary Figure 1.tif]

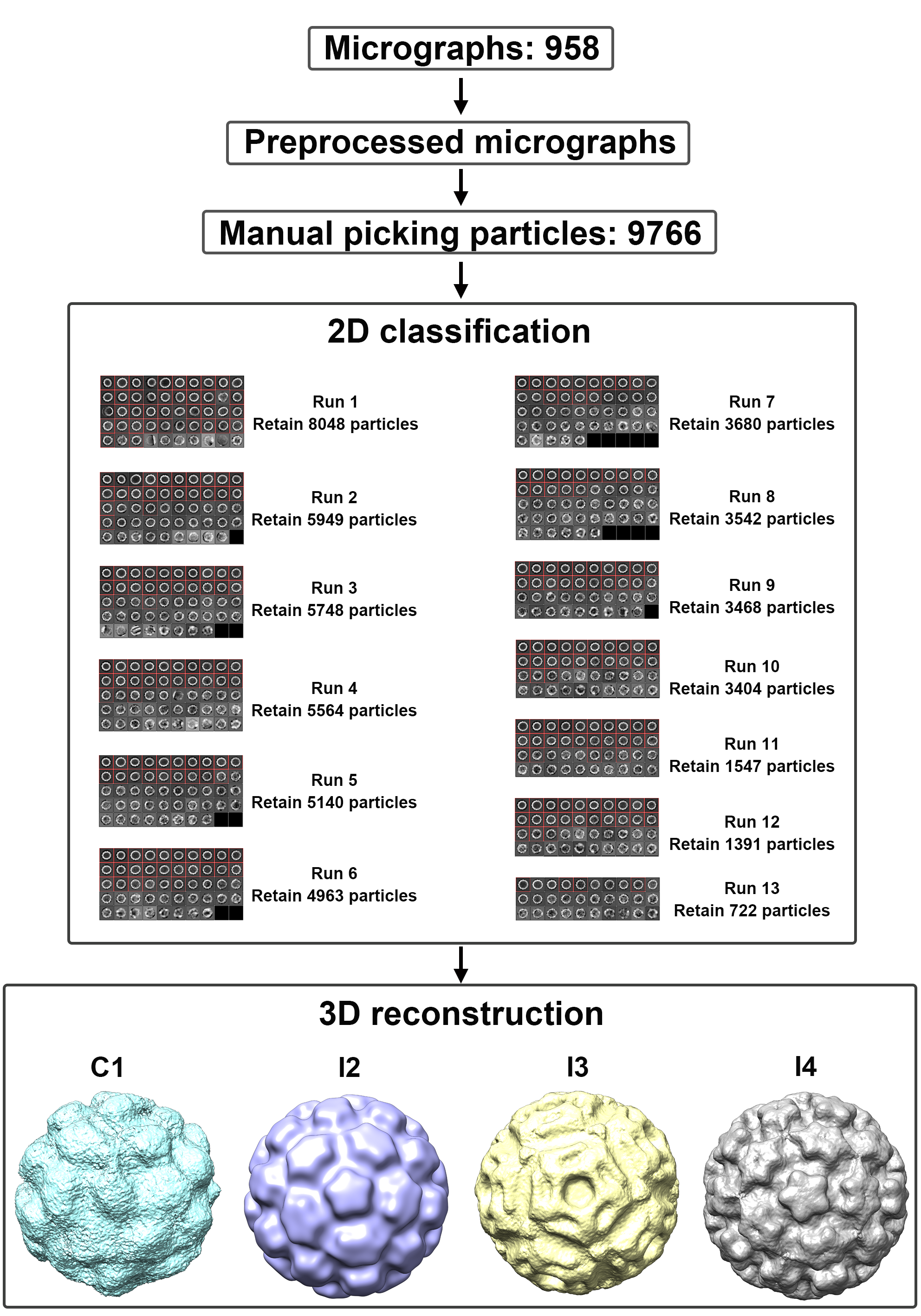

Supplement: Supplementary file 1 [file viruses-10-00667-s001.zip › 0-viruses-382038-supplementary/Supplemenatry files/Supplementary Figure 2.tif]

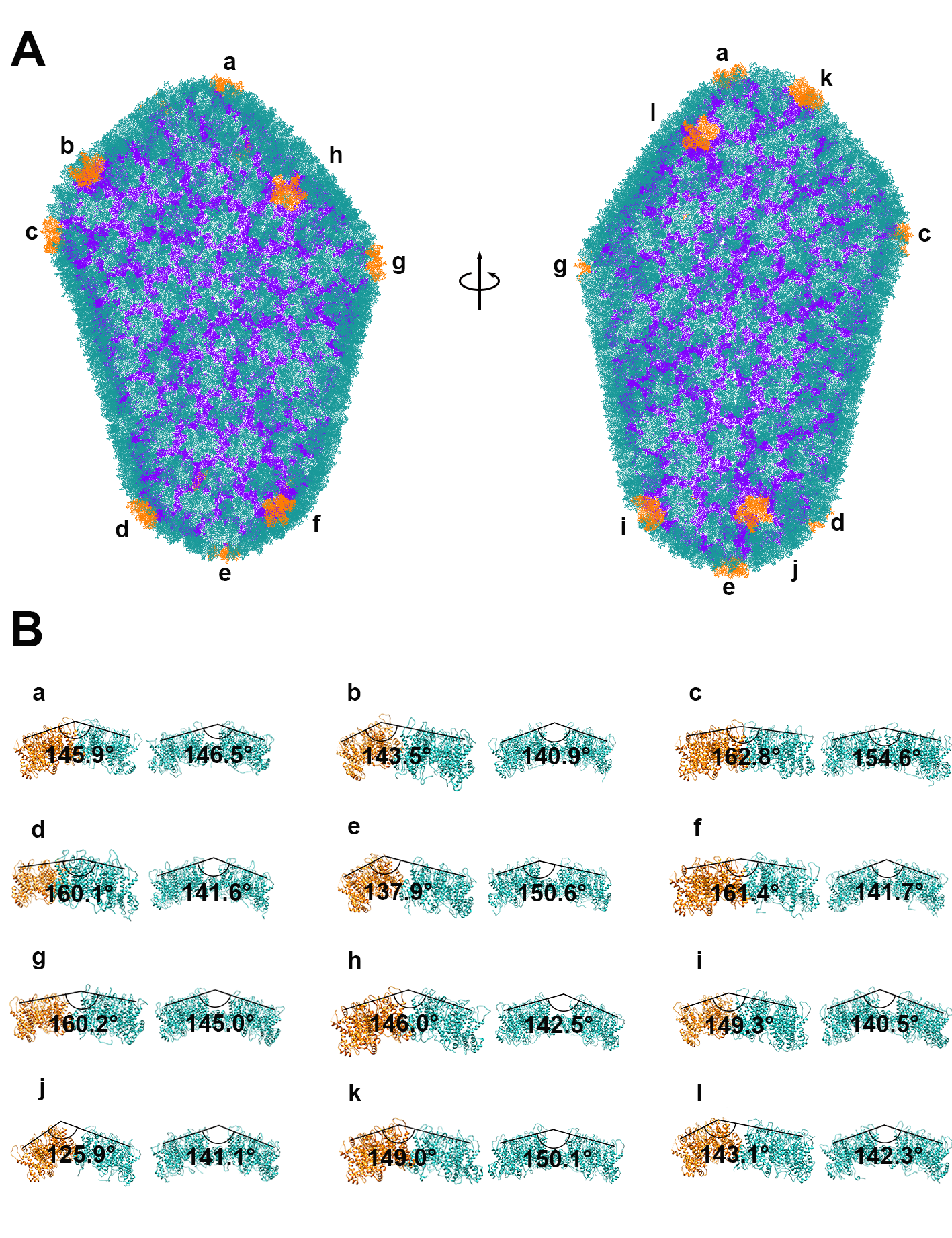

Supplement: Supplementary file 1 [file viruses-10-00667-s001.zip › 0-viruses-382038-supplementary/Supplemenatry files/Supplementary Figure 3.tif]
